# Transformative capacity to build an adaptive society resilience during crisis: evidence from conditional cash transfer/PKH Indonesia

**DOI:** 10.3389/fsoc.2025.1507177

**Published:** 2025-05-07

**Authors:** Keukeu Komarawati, Muhammad Fadhil Nurdin, Wahju Gunawan, R. Nunung Nurwati

**Affiliations:** ^1^Department of Sociology, Padjajaran University, Bandung, Indonesia; ^2^Padjajaran University, Bandung, Indonesia

**Keywords:** transformative capacity, social resilience, conditional cash transfers (CCT), *Program Keluarga Harapan (PKH)*, COVID–19, community resilience, social protection programs

## Abstract

This research study the role of transformative capacity in building an adaptive society’s resilience to crises. This research focus on Indonesia’s Conditional Cash Transfer (CCT) program, *Program Keluarga Harapan* (PKH)/Family Hope Program in the COVID-19 pandemic, Cianjur. Social resilience emphasizes a return to pre-crisis conditions in the traditional view, but this research redefines resilience as the capacity to transform and adapt to new social condition. By analyzing PKH’s impact, this research show that this not only helped beneficiaries cope with crisis but also contributed to longterm changes that enhanced community resilience. This research using a qualitative approach, using interviews and direct observations of PKH beneficiaries in Cianjur. Data collection focuses on understanding the mechanisms through which PKH influenced individuals and families in their response to the crisis. The research examines how financial aid, together with program conditions related to health, education, and social welfare, contributed to behavioral and institutional transformations that strengthened resilience at the household and community levels. Research findings show that PKH played a significant role beside providing financial assistance. PKH also promotes long-term impact by improving access to healthcare, education, and social welfare services. PKH also contributed to changing beneficiaries’ perceptions on poverty and vulnerability, it increased community resilience in every opportunity. The research focus on how PKH facilitated institutional and behavioural transformations that strengthened community resilience during and beyond the pandemic. The study reinforce the importance of policies that support transformative resilience, and go beyond short-term relief to sustainable socioeconomic improvements. PKH’s role suggests that social protection programs should integrate elements that promote long-term resilience, ensuring that vulnerable communities are better equipped to face future crises. These findings offer valuable insights for policymakers in redefining adaptive social protection program and strategies.

## 1 Introduction

The concept of social resilience has evolved significantly, transitioning from its ecological origin to broader applications in social sciences. This evolution has led to several debates, particularly regarding the adaptation of resilience to individual and societal contexts. The simplification and introduction of the concept in social science are considered significantly risky for individuals or actors due to their ability to ignore the surrounding social structure (Olsson et al., 2015). The sociological tradition perceives social resilience beyond the definition as a capacity to revert to earlier circumstances. It was further defined as the practice of directing resources to optimize the positive results for individuals or groups, transforming the status quo, and lessening the effect of unfavorable events in the future (Giddens, 1984: Stones, 2005). Resilience is a double-edged outcome of the reflective and critical process that allows actors to use internal structures after negative events to change the causative external structure conditions. This perspective questions the dominant idea of resilience: agency and structure are separate entities. Instead, resilience emerges from the dynamic interplay between social actors or agencies, which enables individuals and communities to adapt and transform in response to crises.

Indonesia’s experience during the COVID-19 pandemic provides a compelling context for examining resilience. Between 2019 and 2021, the national poverty rate increased from 9.41 to 10.41% due to the pandemic’s socioeconomic impacts. However, by 2023, it had declined to 9.36%, reflecting the government’s efforts to mitigate the crises [BPS (Badan Pusat Statistik), 2023]. The re-expansion of social protection programs, including the Program Keluarga Harapan (PKH), played a critical role in this recovery. These programs prevented a potential spike in poverty to 14% in 2021, reducing its impact by four percentage points (Suryahadi et al., 2021). PKH, Indonesia’s Conditional Cash Transfer (CCT) program, exemplifies resilience by combining aid with behavioral and institutional transformation. The pandemic disrupted multiple aspects of life, forcing vulnerable communities to develop coping strategies to adapt to rapidly changing circumstances. At the community level, social and organizational systems become crucial for handling the crisis and enabling collective action and support. However, these social relations presented a paradox: while strong social ties facilitated coping strategies, they also hindered the implementation of health protocols, highlighting the complexity of resilience during crises (Mawardi et al., 2021). This duality shows the importance of financial but also structural and behavioral transformations in fostering resilience.

PKH serves as a critical component of these community-level resilience efforts, bridging the gap between individual coping strategies and structural transformations. While community social systems played a vital role in navigating the pandemic’s challenges, PKH complemented these efforts by providing a structured framework for resilience building. Through its conditionalities—such as accessing health services, ensuring children’s education, and participating in group meetings—PKH addressed immediate subsistence needs and facilitated long-term behavioral and institutional changes. This alignment of community support systems with programmatic interventions shows how PKH empowered beneficiaries to reconfigure their socioeconomic realities, enabling them to adapt and transform in response to adverse conditions such as those caused by the COVID-19 pandemic.

Using Freire’s critical consciousness and Giddens’ structuration theory, this study frames resilience as a transformative capacity, addressing the root causes of vulnerability rather than merely responding to immediate shocks. It highlights PKH as a “configuration” of agency and structure, fostering behavioral and institutional transformation among beneficiaries.

This research employs a qualitative approach to explore how PKH fosters resilience among its beneficiaries in Cianjur, Indonesia, a region significantly affected by the pandemic. Data was collected over 6 months, from June to December 2023, using semi-structured, in-depth interviews and observations. The study involved 20 informants, including PKH beneficiaries from various cohorts (2015–2021), facilitators, and regulators at the regency level. Informants were purposively selected based on their ability to provide rich and informative data, particularly concerning their coping, adaptive, and transformative capacities during the crisis. Focusing on the interplay between individual agencies and social structures, the study aims to capture the complexity of resilience-building among PKH beneficiaries. This qualitative methodology allows a nuanced understanding of how PKH contributes to transformative capacity, moving beyond immediate recovery to address systemic vulnerabilities and inequalities.

In light of these considerations, this research addresses the following question: “How do Indonesian Conditional Cash Transfer programs assist individuals in escaping poverty and changing the perception of social resilience?” By examining PKH’s role in fostering transformative resilience during the COVID-19 pandemic and preparing them for an unforeseen event ahead of time, this study contributes to adaptive social protection literature and provides actionable policy recommendations for enhancing resilience among vulnerable populations.

### 1.1 Theoretical framework

#### 1.1.1 Program Keluarga Harapan enabling a transformative capacity approach to poverty capacity approach to poverty reduction and social equity

Paulo Freire (1921–1997), a Brazilian educator and philosopher, is best known for his influential work, Pedagogy of the Oppressed (Freire, 1970). His theory of transformative capacity is on the power of public consciousness to drive community change by empowering marginalized individuals and communities to know and understand systemic oppression. In Freire’s transformative theory, several core concepts underpin the process of driving meaningful social change. At the heart of his approach lies critical consciousness, which emphasizes the development of a deep awareness of one’s social reality. Anthony Giddens highlights that individuals (agency) are shaped by and capable of reshaping social structures. Transformative capacity emerges when individuals or collective actors use their agency to challenge and reconstruct established social norms, policies, and power relations. Bourdieu (1979) explains how internalized norms, behaviors, and social capital can be mobilized to challenge social hierarchies. Transformative capacity arises when marginalized groups leverage their social capital to disrupt established power structures.

Marx and Engels (1848) views transformative capacity through the lens of class struggle. Transformation occurs when the proletariat mobilizes collective action to overthrow oppressive systems and redistribute power and resources. Examples of transformative capacity in the modern context are civil rights movements, feminist movements, climate justice movements, and land reform. In the post-modern sociological contexts, transformative capacity includes addressing structural causes of wealth disparity through progressive taxation and social protection programs. Ensuring equal access to technology and digital education reduces inequalities and builds participatory democratic institutions to amplify marginalized voices in policymaking.

In the last decade, Conditional Cash Transfer (CCT) in Indonesia, known as Program Keluarga Harapan (PKH)/Family Hope Program, one of the social protection programs, has been considered an effective policy instrument to improve the lives of the poor. Prior research showed that transfers improved welfare and facilitated human capital accumulation (Fiszbein and Schady, 2009; Ralston et al., 2017). Zhou (n. d.) have shown comparable examples of how conditional cash transfers can foster urban resilience, providing a global parallel to Indonesia’s PKH program implementation.However, the theory underlying the CCT program proved that cash transfers alone could not resolve the causes of poverty (Ibarraran et al., 2017). Levy (2007) stated that the programs needed to incite behavioral changes toward ensuring the accumulation of human capital and the cash provided.

CCT has binding conditions for beneficiaries, called conditionality, compared to unconditional cash transfers. The concept was defined by Ibarraran et al. (2017), p. 35 as behaviors considered favorable to the accumulation of human capital for children of beneficiaries, thereby increasing the ability to generate income in the future and breaking the intergenerational transmission of poverty. This is necessary because social assistance programs are often criticized for causing a moral hazard associated with the decline in insured individuals as a response to the incentives offered by insurance. In Indonesia, CCT/PKH was established by the Regulation of the Minister of Social Affairs Number 01 of 2018 to (1) improve the living standard of beneficiaries, (2) reduce the burden of expenditure while increasing the income of poor and vulnerable families, (3) develop behavioral changes and independence of beneficiaries in accessing health services, education, and social welfare, (4) introduce the benefits of formal financial products and services to beneficiaries, as well as (5) reduce poverty and inequality. The main targets of PKH are the poor and vulnerable families or individuals in the country.

The obligations and conditionalities specified in the PKH scheme are difficult, which include (1) accessing health services for pregnant women, toddlers, and early childhood based on health protocols, (2) accessing education services for school-age children at elementary, junior, and secondary school levels, and (3) attending the monthly meeting. Lucini (2013) showed that community participation is vital to achieving resilience. PKH monthly meetings and participatory facilitation embody this bottom-up involvement in social development. PKH was the most central aspect of the Indonesian Government’s macroeconomic policy package, the National Economic Recovery Program (PEN), during the COVID-19 pandemic. It targeted 10 million poor households in both rural and urban areas, making the CCT program part of the interventions covering the largest number of beneficiaries globally. Meanwhile, the 2020–2022 PEN budget focused on the recovery and survival kits to ensure the effective management of the COVID-19 pandemic and continuous protection of the middle and lower economic groups. This strategic tool was used to motivate 40% of the poor and vulnerable groups to survive through social protection programs. According to Rawlings and Rubio (2005), conditional cash transfers have consistently demonstrated success in improving education and health outcomes, reinforcing the value of PKH’s conditionalities.

Revilla et al. (2018) highlighted the role of targeted social protection programs in enhancing financial inclusion. PKH’s push to introduce formal financial services to beneficiaries is aligned with this trajectory. The programs were necessary because the pandemic affected several aspects of lives, including the economy (Supriatna, 2020), health (Olivia et al., 2020), industry, education, and social sectors (Chaney, 2020; Walsh, 2020). Dagdeviren et al. (2020) argued that social protection must go beyond economic relief and encompass dignity, agency, and structural transformation. PKH’s design with behavioral conditionalities shows an attempt to incorporate this multidimensionality. The economic impact had a multiplying effect on virtually all facets of the individual. Individuals faced threats of business bankruptcy, job loss, reduced income, changing work patterns, and low consumption levels (Gandasari and Dwidienawati, 2020). Moreover, the individual was placed in a state of alienation and mental disorders due to stress and uncertainty (Prime et al., 2020; Brock and Laifer, 2020; Lian and Yoon, 2020), including the potential for death, unemployment, and self-imposed exile, which traumatized individuals psychologically (Abdullah, 2020).

#### 1.1.2 Adaptive society and resilience to navigate change in crisis

The resilience concept has evolved and been applied across various disciplines, including ecology, sociology, psychology, development studies, and social protection. The idea of resilience is to enable individuals and communities to adapt to the crisis’s situation. Holling (1973) introduced the term resilience in ecological systems, explained as the capacity of an ecosystem to absorb disturbances and reorganize while undergoing change, retaining essentially the same function, structure, and feedback. The expansion of resilience to social systems began in 1980–1990. The term became popular in disaster risk reduction (DRR), especially after the Hyogo Framework for Action (2005). Social protection started incorporating resilience-building as an objective in 2010. Brown and Westaway (2011) highlight that resilience is not only the capacity to absorb shocks but also to self-organize and learn, emphasizing the need for proactive adaptation and transformation—principles evident in PKH’s strategic response during the COVID-19 pandemic. The United Nations Development Program (2014) highlighted resilience as a critical element in fostering human development and reducing inequalities. United Nations Office for Disaster Risk Reduction (2015) under the Sendai Framework has positioned social protection as a pillar of disaster risk reduction. PKH’s role during COVID-19 was in direct alignment with this global framework.

A multi-dimensional approach that includes absorptive, adaptive, and transformative capacities has been primarily developed and popularized in the context of resilience theory, with several key contributors and institutions shaping it. Béné et al. (2012) and Bene et al. (2014), in their work on resilience in development and disaster risk reduction, proposed a three-tier framework for understanding resilience.

To understand resilience as the result of absorptive, adaptive, and transformative capacity. Understanding how these capacities interact and contribute to overall resilience is essential. Resilience is not a stand-alone concept but the cumulative result of the interplay between three capacities. Absorptive capacity is the ability to withstand and cope with shocks without significant changes to livelihood strategies or systems. Adaptive capacity is the ability to make incremental adjustments or changes to reduce vulnerability and improve conditions in response to evolving risks or opportunities. Transformative capacity is the ability to create systemic change by addressing the root causes of vulnerabilities and inequalities.

Parsons (1951) developed the AGIL Framework, which includes four key functions: Adaptation, Goal Attainment, Integration, and Latency. Adaptation describes a society’s capacity to adjust to its environment and fulfill its essential needs. Goal attainment refers to a society’s ability to establish and accomplish shared goals and objectives. Integration involves maintaining social cohesion and solidarity among the members of society. Lastly, latency, or pattern maintenance, highlights the society’s ability to preserve and pass down its cultural patterns, values, and norms to future generations.

As adaptive society and resilience, the close definition is disaster management. It is necessary to understand the phases in disaster management and where PKH lies in these phases. Disaster management consists of four stages: prevention, preparedness, response, and recovery. According to Sendai Framework Terminology on Disaster Risk Reduction, prevention is activities and measures to avoid existing and new disaster risks. Preparedness refers to the knowledge and capacities developed by governments, response and recovery organizations, communities, and individuals to effectively anticipate, respond to, and recover from the impacts of likely, imminent, or current disasters. Response is an action taken directly before, during, or immediately after a disaster to save lives, reduce health impacts, ensure public safety, and meet the basic subsistence needs of the people affected. Recovery is the restoring or improving of livelihoods and health, as well as economic, physical, social, cultural, and environmental assets, systems, and activities, of a disaster-affected community or society, aligning with the principles of sustainable development and “build back better” to avoid or reduce future disaster risk. Maclean et al. (2014) emphasized that adaptive capacity is heavily dependent on institutional support. PKH plays this role by enabling access to education and health systems, fostering intergenerational human capital accumulation. Sapountzaki (2007) warned that resilience-building must address spatial and class-based inequalities, a reminder of the importance of ensuring PKH reaches urban and rural poor equitably.

#### 1.1.3 Structuration theory

Structuration theory was introduced by Anthony Giddens in *The Constitution of Society* (1984). The aim is to resolve the long debate related to the determination of factors influencing most human actions between agencies, such as individual needs, wants, and desires, as well as broader structures of individuals, including norms and boundaries (Stones, 2005). Giddens mainly argues that structure is not an ordinary phenomenon developed to shape needs and wants. It is rather socially constructed based on the actions of individuals and groups through a continuous process of renewal or change in implementing daily activities. Therefore, structure frames, which are products of agency and subsequent interactions, can be used in developing social systems. Castel (1995) emphasized that structural disintegration can produce new forms of social vulnerability, which aligns with the rationale behind conditional cash transfers as protective mechanisms to prevent descent into chronic poverty. Campbell L. M. (2005) illustrated how structuration theory applies to local resource management, drawing parallels to how beneficiaries navigate formal and informal systems under PKH.

The theory has been applied extensively in social sciences to different topics, including corporate management (Pozzebon, 2004a; Pozzebon, 2004b), hunting in Africa (Campbell B., 2005), and humanitarian aid intervention (Rodon et al., 2012). However, it has also been criticized by social theorists questioning the argument of Giddens that structure exists “only in a virtual way, as memory traces and as representative rules in certain activities of actors” (Giddens, 1984, p. 256). The critics argued that the position limits the possibility of structure existing in social and material contexts and the form of interactions between individuals (Hodgson, 2007a; Hodgson, 2007b, p. 104). In recent years, sociologist Stones (2005) has attempted to counter this criticism by proposing ‘a quadripartite cycle of structuration’ consisting of four stages explained as follows:

- The external structure forms the structural context social actor’s face at the start of the cycle and is considered a prerequisite for action.
- The internal structure within the actor is divided into two parts. The first is the conjunction-specific or positional structure directed toward the external aspect and formulated based on the actor’s knowledge of a specific action context. The second is habitus, which is explained as the part of social life normally accepted to be just and not questioned again.
- Active actors or actor practices represent the methods adopted by actors to both pre-reflectively and critically use internal structures.
- Outcomes that focus on the change, elaboration, or reproduction and preservation of structural context to facilitate or thwart the goals of the actors.

The strength of Stones’ model is the provision of explanation to structures shaping the means and results of social action while also describing the position during the interaction of social actors.

#### 1.1.4 CAT framework

The Cat framework placed in the logic of sociological structuration theory was used in this research based on the current discourse of contemporary social resilience conceptualized by several authors, including Voss (2008), Lorenz (2013), Obrist et al. (2010), Béné et al. (2012) and Keck and Sakdapolrak (2012). Therefore, understanding social resilience as a whole requires acknowledging the existence of different capacities, including coping, adaptive, and transformative, embodied in four criteria.

The first criterion refers to the response of the individual to risk to differentiate activities before (*ex-ante*) and after (*ex-post*) the crisis. The second criterion, temporal scope, is the intended time horizon, emphasizing a continuum span between agencies based on immediacy, short-term thinking, project-related, and calculative long-term reasons. The third criterion is the degree of change experienced by social structure, while the fourth refers to the outputs or achievements related to the three capacities. The criteria were further used to place each of the resilience capacities in the social resilience matrix as follows:

- Coping capacity is the “reactive” (*ex-post*) (Obrist et al., 2010, p. 289) and “absorb” (Béné et al., 2012, p. 21) actions related to the method applied by individuals to cope with immediate threats through available resources. The rationale is to restore the current level of well-being immediately after a crisis event.
- Adaptive capacity is the “proactive” (*ex-ante*) (Obrist et al., 2010, p. 289) or “preventive” (Béné et al., 2012: 31) actions individuals use to learn from past experiences, anticipate future risks, and adapt their livelihood. Adaptation is directed at gradual change and secures the current state of social welfare in the face of future risks. The main difference between coping and adaptation is the temporal scope of the activity associated with each capacity. Coping addresses tactical agency and short-term thinking, while adaptation focuses on strategic agency and long-term planning.
- Finally, transformative or “participatory capacity” (Voss, 2008; Lorenz, 2013) includes the ability of individuals to access assets and assistance from the wider socio-political arena, such as government and civil individual organizations. It also focuses on participation in decision-making processes and establishing institutions to enhance individual well-being and promote the resilience of individuals against future crises. The main difference between transformation and adaptation is the degree of change and the results implied. Transformation is directed at radical change without the intention of securing but to improve the welfare of individuals in facing current and/or future risks. It also includes topics related to progressive change and development.

## 2 Methodology

This research was conducted in the province of West Java in Cianjur Regency, and the PKH beneficiaries were used as the population. The preference for Cianjur was because the regency was significantly affected by COVID-19, leading to the termination of personal contracts (PHK) of laborers and a layoff of several workers (Nurhidayati and Pandin, 2021). During the pandemic, the Community Development Index was 65.36, placing the regency in the extreme poverty category and last rank, at 27th, among all HDIs in West Java [BPS (Badan Pusat Statistik), 2021].

The condition of PKH beneficiaries during the COVID-19 pandemic and the response to the crisis were investigated using qualitative methods. The aim was to describe and interpret the social resilience model—understandably quite complex—developed by PKH beneficiaries during the pandemic. Moreover, the research also attempted to present a comprehensive overview and description of an issue based on the field phenomena observed directly by research informants.

Twenty female PKH beneficiaries in Cianjur Regency participated in the six-month data collection period from June to December 2023. The PKH cohorts ranged from 2015 to 2021. The selection of informants was based on their capacity to supply useful information and their active participation in the family’s economic function. To qualitatively investigate the level of PKH beneficiary social capital, interviews were also done with those directly involved in PKH management and other significant players, both formal and informal. Key players outside the program also helped identify issues with PKH beneficiaries’ social resilience capabilities. The coping, adaptability, and transformational capability criteria were used to choose informants. Since not all districts matched these requirements, three neighboring sub-districts on the Cianjur area map were randomly selected to provide a more representative study by accounting for social, economic, and cultural similarities.

The qualitative method was applied from the data collection process to analysis. The expectation was to identify and resolve the complexity of the problems faced by PKH beneficiaries after the pandemic. This was intended to be achieved by examining social resilience through the dimensions of social capital owned by PKH beneficiaries as individuals. A total of 20 informants, including regulators, PKH beneficiaries from 2015–2021 cohorts, and facilitators, were used, as stated in the following Table 1. Moreover, the strength of social capital possessed by PKH beneficiaries was assessed using semi-structured, in-depth interviews conducted with key actors, both formal and informal, as well as those that directly manage PKH in the individual.

**Table 1:** Research participants.

Research Participants	Information	Numbers	Total
Regulator	Representation of the Ministry of Social Affairs	1	2
	Representation of implementer at regency level	1	
PKH’S Facilitator	Facilitator of the PKH Program assisting KPM in withdrawing, using assistance, and attending monthly meetings	3	3
PKH Beneficiaries	Cohort 2015	2	15
	Cohort 2017	6	
	Cohort 2018	4	
	Cohort 2019	1	
	Cohort 2020	1	
	Cohort 2021	1	
TOTAL			20

The study analyzed interviews using NVivo 9.2 software to investigate the transformational abilities of PKH recipients in Cianjur, West Java. Themes such as program knowledge, informant experiences, crisis management, and agency transformation were used to group the data. The four steps of an inductive approach—field observations and data classification, topic reclassification, open, axial, and selective coding, and story production using visual pattern representations—refined presumptions and investigated phenomena. In the end, the qualitative approach was strengthened, and significant conclusions were drawn using the CAT framework to evaluate social resilience and secondary data, which was further supported by diagrams.

## 3 Results

### 3.1 Transformative capacity in using PKH interventions to assist individuals in becoming social agents capable of overcoming obstacles

The field research showed that PKH beneficiaries were aware of their rights and responsibilities. The focus was on the first participation, the amount received as assistance, what the money was used for, where the assistance was received, and attendance in monthly meetings. The informant gladly shared information presented as follows.

*“I have been a PKH participant for about 7 years. The first time I received assistance was when my second child was in elementary school, then I used the first assistance as a business capital.” (Informant On, 2023).*

The interesting observation was that Mrs. On used the assistance for business capital, which was considered a very progressive step at the time because first recipients were always reminded to prioritize the school and health needs of the children. The information also showed a good memory, as I remember the first time the assistance was disbursed. Informant On admitted that assistance was first withdrawn at the Post Office, while Mrs. Ni recalled it was at the BRI Brilink Agent and Mrs. Di at a BRI ATM. The Ministry of Social Affairs, as the implementer of PKH since 2016, expanded the assistance distribution institutions, which initially only included PT Pos Indonesia, to include state-owned banks such as BNI, BRI, Mandiri, and BTN. In Cianjur Regency, the appointed distribution institution apart from PT Pos is Bank BRI.

Another responsibility observed to have been fulfilled and remembered effectively was monthly meeting attendance. The response of Informant Ni (2023) to the question related to the frequency of attendance is presented as follows:

*“If I’m not mistaken, every month (attending monthly meetings) since I registered for PKH.”*

The content mostly remembered by informants On and Di was focused on health, education, nutrition, stunting, and money management. This was observed from the acknowledgment of Informant On that the resources for financial management were exactly the support the company required. Despite not having completed elementary education, she acknowledged her ability to easily compute and recall sales financial data. The justification shows the transformative capacity of beneficiaries through a solid understanding of PKH. The results were subsequently simplified in line with the knowledge of beneficiaries in Table 2.

**Table 2:** Knowledge of the program.

Aspect	Subpoint	Description
Knowledge about the Program	Cohort participation	Remembering the participant cohort effectively, the knowledge of the program is explained very well with good memory and high enthusiasm.
	Rights and obligations as a participant	Beneficiaries can comprehensively explain their rights and obligations.
	Amount of assistance and withdrawal	Assistance was allocated effectively at different values but with a focus on the relevance to the program’s objectives. Assistance was provided for business capital to improve the family economy.
	Use of assistance	Diligently participating in monthly meeting activities and having several reminders on the materials presented on general knowledge, stunting, health, nutrition, childcare, children’s education, schools, and starting a business in memory.

Source: own elaboration.

Using Paulo Freire’s transformational capacity theory, the research findings on the perceptions and behaviors of PKH beneficiaries may be analyzed. Freire highlighted the significance of critical awareness in enabling people to confront oppressive circumstances. In this case, Informant On, who benefited from the initial business capital support, showed transformational potential by using the help to empower himself. This illustrates how the PKH program serves as both a source of financial aid and a change catalyst, enabling users to use resources to enhance their well-being. According to Anthony Giddens, individual agency is demonstrated by how receivers employ assistance to transcend their status as passive recipients. Similar to the tiny business that Informant On created, they actively use help to make changes in their life. This also illustrates how the PKH program’s social structure encourages social change by allowing people to exercise their agency. Participation in monthly meetings by beneficiaries demonstrates a comprehension of their responsibilities, which fosters group awareness and improves each person’s ability to obtain social services. These results demonstrate that PKH serves as a social transformation instrument that enables recipients to use assistance to bring about more significant change in their lives. This demonstrates the applicability of theories of class struggle, social capital, agency, and transformational capability in the framework of contemporary social policy.

#### 3.1.1 Experience from the PKH program is useful when facing difficulties

PKH assistance was used effectively, specifically for the education and health needs of the children. Informants Ni, On, and Di admitted that the assistance received was very helpful in sustaining the family’s economy during the pandemic. Moreover, the experience garnered in managing assistance was applied to prioritize needs over wants during difficulties. For example, the family’s diet was prioritized when income decreased during the pandemic. This was achieved by sustaining meals three times a day while working around the daily menu.

The results further showed that all beneficiaries had a card and understood the need for constant updating as a duty to safeguard assets. The trend was observed from the responses provided, such as the information presented as follows:

*“Since it is a personal right and should not be held by others, it can be used to disburse aid” (Informant Ni, 2023).*

The card provides beneficiaries access to official financial institutions, boosts confidence, and allows more opportunities to deal with other individuals. Informant Di stated that she used non-cash transactions to manage her small company and discerned between legitimate and phony evidence of transfer. Therefore, she was not concerned about completing transactions during the pandemic due to the convenience provided by banking facilities. The account owned by Di was used to withdraw aid and conduct other financial activities to support the business.

Attending monthly meetings is perceived as a method of improving skills and expertise. During the pandemic, financial management became a very useful skill, specifically in managing money to be used as business capital. The more interesting aspect was the report of Informant Di, which stated that the regular meeting facility opened up opportunities to expand the networks needed to develop businesses.

*“…there are several groups and opportunities to offer merchandize”* (Informant Di, 2023).

PKH increases the confidence of participants in relation to decision-making. For example, the assistance provided was based on a joint decision, with each member conveying information related to education, health, and productive purposes. The partners were also supportive, as observed by the wives accompanying the husbands during the interview and reinforcing the information provided. Moreover, the informants exhibited a very strong self-confidence every time during the interview and answered questions frankly and enthusiastically. The wives also became breadwinners due to the loss of jobs during the pandemic, while husbands assisted with the running of the businesses. This was observed from the response of Informant On that the daily activities and tasks were shared with the husband based on the agreement of both partners.

*“Si Aa (My husband) helps with packing when there are orders”* (Informant Di, 2023).

The explanation showed that the PKH experience allowed the participants to leverage the transformative capacity when faced with difficulties. These results were simplified and presented in the following Table 3.

**Table 3:** Experiences as PKH beneficiaries.

Aspect	Subpoint	Description
Experiences as PKH Beneficiaries	Use of assistance	Assistance was used according to the objectives of the program, and the application of a priority scale in the process of spending was made. The majority used the assistance to meet food needs during the pandemic, and efforts were made to keep the business running using the assistance, and they graduated from the program.
	Holding KKS	The participants understood the card needed to be held personally, the importance of holding the card as an obligation to protect assets, agents or ATMs were designated places to collect assistance, and experience in holding a card was used to develop self-confidence and to eventually know formal financial institutions, the opportunity to conduct other financial transactions.
	Follow and Apply Knowledge from Monthly Meeting	Attendance of monthly meetings was a resource to improve skills and opportunities required to move up a class. The materials provided during the pandemic were remembered and applied; the meeting provided opportunities to expand networks needed to develop businesses; financial planning modules about nutrition and parenting patterns were remembered for subsequent application to survive; the food menu of the children was sustained despite the difficulties; and business ideas were generated.
	Decision-making	Very confident in making decisions, specifically through the usage of first assistance as capital, and dominant in decision-making compared to couples that enjoyed support from their husbands and family.

Source: own elaboration.

### 3.2 Transformative capacity assisted PKH beneficiaries in escaping poverty

#### 3.2.1 PKH beneficiaries as transformative agents

The COVID-19 pandemic challenged the capacity of beneficiaries to deploy knowledge, technology, and economic resources to withstand shocks and pressures. As the customary response of KPM PKH to pandemics, the group started with fear and unrest before surrendering to the Almighty, as observed in the following statement:

*“Just ask for forgiveness, it will not get to anything, face the divine and pray, kitu we kasaha deui lumpatna (that’s why we started the business again)” (Informant On, 2023).*

The informant started business again during the crisis after waiting for a week to identify the impact of the pandemic. The key to the effective response to the challenges was associated with the presence of a supportive spouse during difficult times. The ability of the participants to mobilize economic, technological, and information resources to withstand shocks and pressures was tested during the COVID-19 pandemic. PKH beneficiaries perceived the pandemic as a common occurrence by showing some anxiety and restlessness before surrendering to the Almighty.

The economic impact of losing the livelihood of the husbands was alleviated by social assistance received from PKH. However, the assistance was insufficient, leading to the decision of an informant to sell assets such as gold jewelry that were considered not to have significant value.

*“Even though it is only 1 g, I will sell the jewelry when I’m in a pinch for daily needs” (Informant Di, 2023).*

The response further showed that some borrowed from relatives, specifically parents. However, Mrs. On did not stop selling her products due to social restrictions because she had exhausted her savings.

*“muhun teu aya (income) seep pisan so akapsa we sell deui well, there was no (income), so we needed to sell again “(Informant On, 2023).*

The COVID-19 pandemic became the momentum for the success of the business Informant On pioneered. She initially sold fried foods and expanded to a grocery store that was slowly equipped with daily necessities. During the pandemic, the shop was visited by several nearby individuals due to its proximity to the settlement. Meanwhile, informants Di and Ni had previously changed from selling small food to returning to the business during the pandemic. The sales stopped, forcing the informants to adopt a pre-order system via the WhatsApp group and a slow effort to use social media as the platform to sell due to the increasing need.

Informant Di started a business through a WhatsApp group before the pandemic but attempted to join a marketplace during the crisis using the feature to sell the goods live. Finally, she joined an agency to make live sales and expand the market to different parts of Indonesia and even neighboring countries. The informant initially sold snacks but changed to clothes and joined different WA sales groups. She did not produce food and clothes, leading to smaller profits despite a larger market share and faster money turnover. Informants Ni and Di have joined several agents supplying merchandize in large quantities. The trend showed that the PKH beneficiaries became transformative agents to survive and improve their welfare. Therefore, the results related to this section are simplified in the following Table 4.

**Table 4:** Responses of beneficiaries to crisis.

Aspect	Subpoint	Description
Beneficiaries as transformative agents	Response to crisis	The participants understood the impact of the pandemic late, felt slightly stressed and anxious, and increased their attitude toward prayers. Efforts were made to find solutions to the difficult times with the partners.
	Strategy for meeting needs	The food menu was changed through sufficient money, the selling of assets, and loans from parents and relatives. Efforts were made to sustain the business and explore other opportunities.
	The source system	Partners, parents, relatives, and social assistance, as well as loans from formal financial institutions, business opportunities from relatives and neighbors, and the expanded reach provided by online platforms.
	Self-adjustment agency: work experience, efforts to form a business or trade, desire to expand the business, collaboration with large business actors, innovation	Most beneficiaries had experience in the formal sector, started a trading business before receiving PKH assistance, and had the required ability. Some beneficiaries decided to sell online, which was profitable due to the compulsory social restrictions. Pre-order sales, live streaming, and reselling systems were adopted. Beneficiaries were optimistic about progress, as evidenced by the exit from the program and the desire and efforts to expand the business. Rapid growth was recorded after implementing online marketing. Beneficiaries joined an agency that supported live sales and expanded the market outside Indonesia. The focus was on food and fashion, which led to the participation of several WhatsApp sales groups. Suppliers were agents found online and individuals who consigned goods to be sold. The market was expanded through online media such as WhatsApp groups and marketplaces, and the business was diversified by exploring available opportunities.

Source: own elaboration.

PKH beneficiaries’ resilience can be understood using Parsons’ AGIL framework. Their capacity to adapt to pandemic-related societal and economic shifts, including moving to an Internet business model, is an example of adaptation. Their success in preserving family financial security through small businesses is evidence of goal attainment. Social interactions within the community, such as exchanging resources and information, lead to integration. The manner in which beneficiaries emphasize health and education ideals even amid trying circumstances is an example of latency, or the maintenance of cultural norms. The PKH program supports the recovery and readiness stage of disaster management as defined by the Sendai Framework. Poor families that lost their jobs during the epidemic can improve their financial situation with the support of PKH’s social assistance. Furthermore, beneficiaries are better prepared to handle comparable difficulties in the future because of the knowledge they receive from monthly PKH meetings on financial and health management topics. In keeping with the idea of “building back better,” the resilience developed by the PKH program enables families to recover and fortify their ability to withstand threats in the future.

PKH beneficiaries were compelled by the challenging circumstances to use the skills possessed and organize resource systems to survive. This led to the implementation of efforts to develop profitable inventions to influence the wellbeing of families and the environment, as evidenced by the increase in revenue. Successfully transformed beneficiaries decided to leave PKH due to the increment in income, which covered basic needs and provided employment opportunities for families and relatives. Most joined the 2015 cohort and decided to leave in 2022, while the 2017 cohort left in 2021 and the 2019 cohort in 2023. It was observed that the participation period did not necessarily influence the transformation of KPM. Meanwhile, the combination of knowledge and experience as PKH participants, the formal and informal sectors, efforts to start a small business, the desire to expand the business, collaboration with large business actors, and innovation led to increased welfare.

## 4 Discussion

Transformation is the change in the physical, social, or economic conditions beyond the context of protection to the elevation of the actors, in this case PKH beneficiaries, to a state of social mobility. Beneficiaries’ efforts in dealing with the crisis were the changes and transformations in the structural context existing before the occurrence of the negative event. These were observed from compliance with the necessary obligations as PKH beneficiaries, knowledge and experience acquired, difficulties associated with the crisis, and maintaining the conditions to survive and increase welfare in the post-crisis period.

**Figure 1:**
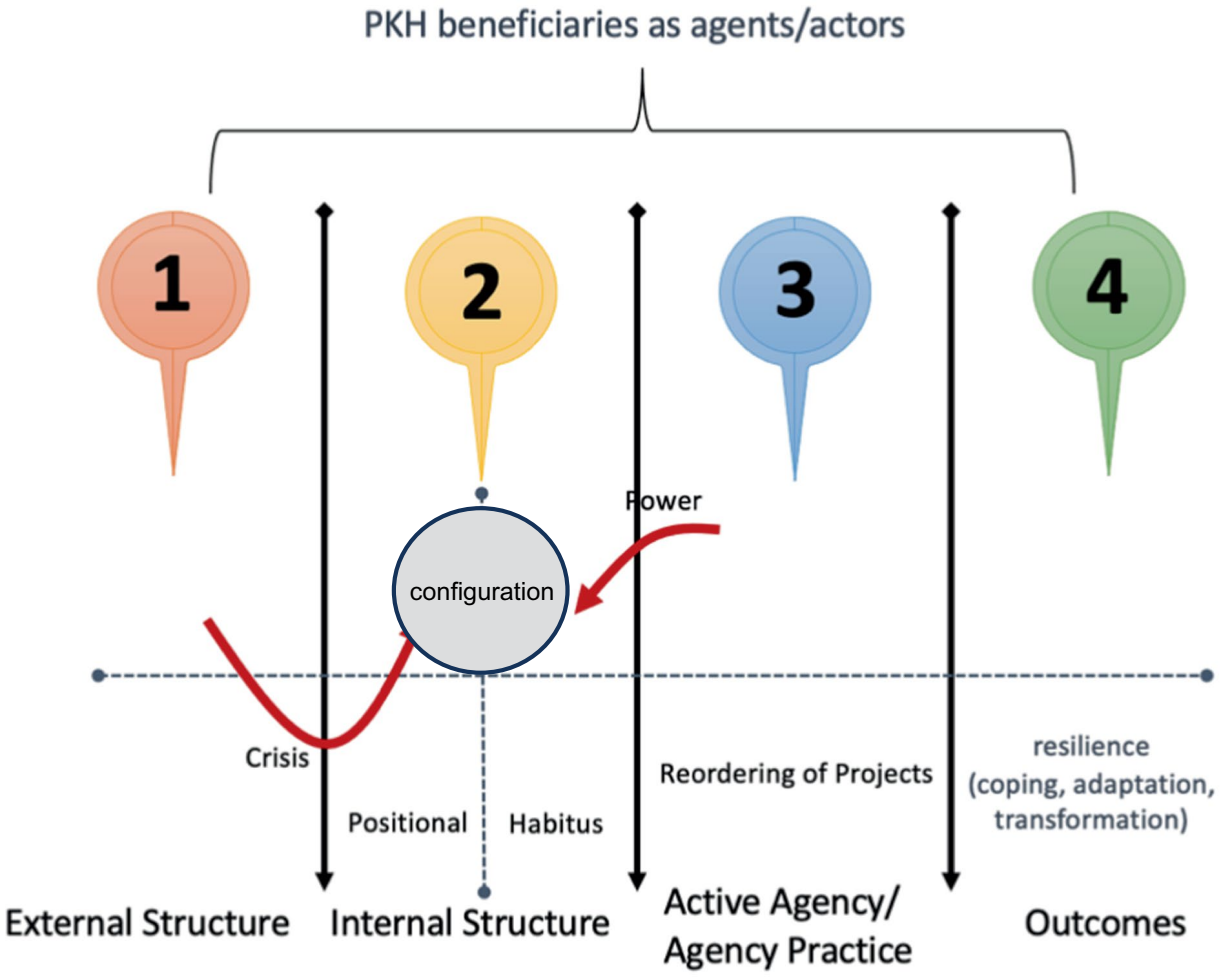
Strategic orientation of actors after the shock or crisis. Source: own elaboration.

The ability of beneficiaries, as social actors, to respond positively to incoming shocks can be explained through Stones’ theorization (2005: 85). This was achieved using the four-stage structuration cycle model, basically designed to consider the responses of actors to recover from or reduce negative events in the form of shocks to crises as presented in Figure 1.

Figure 1 shows that negative events, such as the COVID-19 pandemic and subsequent impacts capable of triggering a response later, are due to the external structure, as presented in Stage 1. The idea of external structure considers both physical events and social circumstances capable of turning the situation into a disaster. In this context, the emergence of the COVID-19 virus caused a pandemic worldwide with subsequent impacts on health, education, and social aspects considered external to the actors. Most beneficiaries were significantly affected by the pandemic, specifically regarding livelihoods.

A negative event can affect actors in two main ways. The first is the impact on the internal structure, as presented in Stage 2, specifically the positional aspect, because the tendency is to act shaken and potentially confront the habitus. PKH beneficiaries are positionally low-income families protected with assistance by the government before the shock. The pandemic led to hardship, which caused stress, and subsequent responses were strongly influenced by religious beliefs, knowledge, and experience as PKH participants, as well as previous life situations or habits, as reported during the field research. The second emphasized the ability of the negative event to hinder the progress of actors toward fulfilling goals initially set, leading to reprioritization in organizing action of Stage 3. The situation led some actors to use the internal structure in new and critical ways to intervene and challenge the patterns of meaning, behavior, and distribution of resources formed in the environment. This process of internal intervention is symbolized in Figure 1 as the ‘Power’ arrow.

Beneficiaries with transformative capacity experienced a series of understandings and accepted the crisis, leading to continuous efforts to find solutions. The assistance provided in the form of money was considered very useful in reducing expenses. The next action was re-designing food expenses and daily needs by regulating eating patterns and menus. Stage 4 produced two main outcomes, including the resilience of agents or actors, which was assessed through the CAT framework in coping, adaptation, and transformation (Keck and Sakdapolrak, 2012; Bene et al., 2014).

The successful mobilization of resources by KPM was observed to be resilient based on the theory of resilience related to the four-cycle structuration model in Figure 1. The ability of a resilient entity, such as an individual or group, to blend and organize strategic orientation is associated with the transformative capacity. This was observed from the configuration results, which were in the form of the PKH condition before the negative event. The explanation is further presented in the following figure.

**Figure 2:**
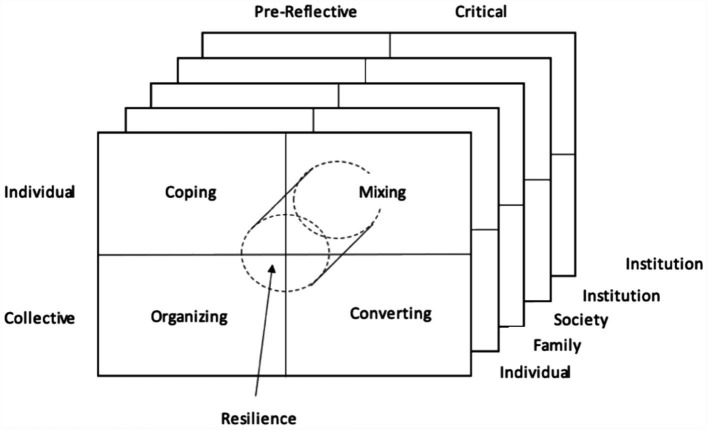
Strategic orientation of actors after the shock or crisis. Source: own elaboration.

The resilient entity, such as an individual or group, can mobilize all the strategic orientations, as reflected by the placement of resilience space at the center of the matrix in Figure 2. There is also the possibility of becoming resilient at several scales at once (Schoon, 2006), with families or individuals acting individually and collectively, as well as in the broader institutional and socio-historical contexts. This shows that structures can shape both the means and ends of efforts implemented by actors to understand new post-impact environments after the occurrence of a negative event. The process is necessary to determine the reasons for the negative events, the subsequent impact on the internal structures of actors, and the strategic orientations and resilience outcomes.

Most beneficiaries had work experience in formal and informal sectors as traders, factory workers, and part-time teachers with below-average incomes. Trading was the main skill, as observed from the responses of all three who had trading experience before becoming PKH assistance recipients. The individuals started businesses by selling one particular type of product using traditional sales methods and experienced different forms of failures. The business has eventually grown to include more than one type of product.

Each KPM improved the types of businesses and marketing strategies as part of the innovation or new methods developed at the critical point. Informant On was loyal to traditional marketing methods but expanded from selling fried foods and drinks to grocery stores equipped with daily necessities at a strategic location to cater to individuals in the surrounding environment. Meanwhile, informants Ni and Di expanded through online media such as WA groups and marketplaces using features that allowed live marketing in order to penetrate the global market and access several agents supplying different types of goods.

The policy implemented by the Indonesian government, known as PKH, provided a framework for distributing and using resources among impoverished groups identified as program participants. This includes access to financial aid to reduce the financial burden, healthcare and education as fundamental services, and monthly meetings that serve as a forum for information sharing and internalizing behavioral modifications. The framework aligns with the program’s goal, which is to improve the quality of life for beneficiaries. The development of resilience was identified as the result of the CCT scheme designed to protect disadvantaged individuals from shocks and social hazards by providing social benefits before the attacks (Schafer et al., 2009).

## 5 Conclusion

In conclusion, the production of resilient beneficiaries was not the official and written purpose of the program, but the importance of resilience was found to be scattered throughout the implementation phases. The tendency was more prevalent in post-COVID crises, specifically through the motivation of PKH recipients to leave the status after achieving a higher economic status. During the pandemic, beneficiaries of the Family Hope Program (PKH) showed remarkable endurance. Their objectives of preserving family financial stability through small enterprises and fostering social integration through community interaction were accomplished as they were able to adjust to social and economic changes. Despite hardship, they also maintained cultural standards for education and health. PKH assists low-income families who have lost their employment in improving their financial circumstances during disaster management’s recovery and preparedness stages. In accordance with the idea of “building back better,” beneficiaries get information through frequent PKH sessions that enable them to not only recover but also strengthen their defiance of potential dangers.

The trend showed the possibility of using structuration to analyze the influence of transformation on the agency and relevant social-economic structure. Keck and Sakdapolrak’s (2013) theory states that the difference in social resilience framework is based on the method applied, including an actor-oriented perspective such as the KPM PKH used in this research. This showed that social resilience, defined as transformability, was important in determining the potential direction for development, specifically concerning poverty alleviation. However, successful implementation significantly depended on the configuration of the internal structure of actors through policies that ensured the transformation dimension was provided as a public good. The results also showed the need to perceive social resilience as a concept capable of assisting in escaping a crisis and addressing the issue of poverty. This was supported by the observation of Mu (2020) that the provision of opportunities could assist in reducing poverty.

## Data Availability

The original contributions presented in the study are included in the article/supplementary material, further inquiries can be directed to the corresponding author.
